# Comparative analysis of extracellular proteomes reveals putative effectors of the boxwood blight pathogens, *Calonectria henricotiae* and *C. pseudonaviculata*

**DOI:** 10.1042/BSR20203544

**Published:** 2021-03-05

**Authors:** Xiao Yang, Michael B. McMahon, Sowmya R. Ramachandran, Wesley M. Garrett, Nicholas LeBlanc, Jo Anne Crouch, Nina Shishkoff, Douglas G. Luster

**Affiliations:** 1 United States Department of Agriculture (USDA), Agricultural Research Service (ARS), Foreign Disease-Weed Science Research Unit, Fort Detrick, MD, U.S.A.; 2 Oak Ridge Institute for Science and Education, ARS Research Participation Program, Oak Ridge, TN, U.S.A.; 3 USDA, ARS, Animal Biosciences and Biotechnology Laboratory, Beltsville, MD, U.S.A.; 4 USDA, ARS, Mycology and Nematology Genetic Diversity and Biology Laboratory, Beltsville, MD, U.S.A.

**Keywords:** Buxaceae, computational biology, Cylindrocladium, ornamental diseases, proteomics, secretome

## Abstract

*Calonectria henricotiae* (*Che*) and *C. pseudonaviculata* (*Cps*) are destructive fungal pathogens causing boxwood blight, a persistent threat to horticultural production, landscape industries, established gardens, and native ecosystems. Although extracellular proteins including effectors produced by fungal pathogens are known to play a fundamental role in pathogenesis, the composition of *Che* and *Cps* extracellular proteins has not been examined. Using liquid chromatography-tandem mass spectrometry (LC-MS/MS) and bioinformatics prediction tools, 630 extracellular proteins and 251 cell membrane proteins of *Che* and *Cps* were identified in the classical secretion pathway in the present study. In the non-classical secretion pathway, 79 extracellular proteins were identified. The cohort of proteins belonged to 364 OrthoMCL clusters, with the majority (62%) present in both species, and a subset unique to *Che* (19%) and *Cps* (20%). These extracellular proteins were predicted to play important roles in cell structure, regulation, metabolism, and pathogenesis. A total of 124 proteins were identified as putative effectors. Many of them are orthologs of proteins with documented roles in suppressing host defense and facilitating infection processes in other pathosystems, such as SnodProt1-like proteins in the OrthoMCL cluster OG5_152723 and PhiA-like cell wall proteins in the cluster OG5_155754. This exploratory study provides a repository of secreted proteins and putative effectors that can provide insights into the virulence mechanisms of the boxwood blight pathogens.

## Introduction

Boxwood blight is a destructive disease of *Buxus* species [1,2]. Since the first observation of this disease in the United Kingdom in 1994 [3], it has spread worldwide, disrupting boxwood nursery production, disfiguring plantings in landscapes and gardens, and reshaping natural ecosystems. Although fungicide applications can be effective in reducing disease development [1,4], there is no cure for boxwood blight. Durable host resistance may be the only solution for the boxwood industry.

Boxwood blight is caused by two distinct fungal species in the genus *Calonectria*, *C. henricotiae* (*Che*) and *C. pseudonaviculata* (*Cps*). The geographic origin of these pathogens is unknown. The known invasive spread of *Cps* is throughout Eurasia [5–15], North America [16–19], and New Zealand [20], while *Che* has been found exclusively in Europe [21–23]. In addition to the different geographic distributions, these two species are distinguished by genetic and phenotypic features. *Che* isolates exclusively possess the *MAT1-1* mating-type idiomorph, whereas *Cps* isolates possess *MAT1-2* [23]. Phenotypically, isolates of *Cps* were found to be more thermosensitive and intolerant to the tetraconazole fungicide than *Che* isolates [21]. The presence of a *CYP51A* pseudogene and lack of a functional *CYP51A* paralog in *Cps* may be responsible for its higher susceptibility to tetraconazole [24].

Fungi produce many extracellular proteins that are integral to cell structural and regulatory processes such as nutrient acquisition, cell wall assembly, defense, and interactions with abiotic environments and other organisms [25]. Within the extracellular fungal proteome, a collection of N-terminal signal peptide (SP)-containing proteins is secreted through the endoplasmic reticulum (ER) and Golgi apparatus in the classical secretion pathway [26,27]. A small number of proteins without an SP are secreted through cargo proteins or other unknown mechanisms in the non-classical secretion pathway [26–30]. Among fungal secreted proteins (collectively referred to as the secretome), many proteins function as enzymes to carry out hydrolytic, proteolytic, glucanalytic, and a variety of cleaving reactions [27,28,31]. Secretomes of pathogenic fungi often consist of small (<300 amino acids), cysteine-rich proteins [32]. These small secreted cysteine-rich proteins (SSCPs) that function to promote infection and suppress plant defenses are called effectors.

Fungal plant pathogens secrete effector proteins that can function either in the plant apoplast or be translocated to the host cell cytoplasm. Identification of fungal effectors can provide essential clues into their virulence strategies, as apoplastic effectors are known to counteract apoplastic plant proteins involved in defense responses, while non-apoplastic or cytoplasmic effectors interfere with plant cell signaling, metabolism, and immunity [33,34]. Although fungal effectors lack consensus sequence motifs that are present in bacterial and oomycete effectors, most identified fungal effectors are SSCPs [27,28,35,36]. This feature can be used to predict novel effectors from fungal secretomes. Other than 207 SSCPs and potential effectors identified from genome and transcriptome data of *C. pseudoreteaudii* infecting *Eucalyptus* [35], little is known about effectors of other *Calonectria* species, specifically the boxwood blight pathogens.

In addition to extracellular proteins, cell membrane proteins of fungal pathogens have critical roles in the cell structure and fungus–host interaction. For example, *Aspergillus fumigatus* Af293 employs cell membrane proteins for transmembrane transportation, biosynthesis, cell wall organization, and stress- and drug-induced responses [27]. Transcriptomic analysis revealed that cell membrane proteins produced by *C. pseudoreteaudii* are responsible for the transmembrane transportation of degradation and detoxifying enzymes [35]. While many high-throughput studies have excluded cell membrane proteins from their secretome analyses [28,31], others have reported them along with or as part of the secretome [27,37,38].

To date, no work has been done to identify effectors of the boxwood blight pathogens. However, it is likely that *Che* and *Cps* secrete effectors to suppress host defense and facilitate infection. Therefore, a comprehensive analysis of the extracellular proteins and putative effector candidates of *Che* and *Cps* is warranted to aid the understanding of the interactions between these pathogens and their hosts and environments. In the present study, we analyzed proteins produced by *Che* and *Cps* using liquid chromatography-tandem mass spectrometry (LC-MS/MS). An array of bioinformatic prediction tools was applied to identify extracellular proteins and putative effectors in both classical and non-classical secretion pathways and cell membrane proteins of the two boxwood blight pathogens. These proteins provide targets for further dissection of the biology and pathogenesis of the boxwood blight fungi and potential avenues for developing disease management measures.

## Materials and methods

### Fungal isolates

Four isolates of *Che* and *Cps* were used to produce proteins for mass-spectrometry (Table 1). Isolates were maintained as microsclerotia on sterile GelAir Cellophane Support sheets (Bio-Rad Laboratories, CA, U.S.A.) covering the surface of glucose-yeast extract-tyrosine (GYET) agar [39] for long-term storage. To produce actively growing cultures, microsclerotia-infested cellophane sheets were cut into small squares (approximately 5×5 mm) and placed on to fresh GYET agar at 20°C to facilitate sporulation of microsclerotia and expansion of colonies.

**Table 1:** Isolates of *Che* and *Cps* used in the present study

Species	Isolate	Origin		
		Host	Location	Year
*Che*	JKI 2106	*Buxus* sp.	Germany	2007
	NL009	*B. sempervirens*	The Netherlands	2011
*Cps*	NC-BB1	*B. sempervirens* ‘Suffruticosa’	North Carolina, U.S.A.	2014
	CBS114417	*B. sempervirens* ‘Suffruticosa’	United Kingdom	1999

### *In vitro* production and sample preparation of fungal proteins

Seven to nine mycelial plugs (6 mm in diameter) were taken from the actively growing margin of a 30-day-old culture of each isolate using a sterile cork borer. Fifteen to eighteen plugs of two isolates belonging to the same species were transferred to a 10-cm Petri dish containing 20 ml of sterile MES (2-(N-morpholino)ethanesulfonic acid) buffer (10 mM, pH 6.5). Ten replication Petri dishes were prepared for each species. Petri dishes were incubated in a dark incubator set at 25°C for 3 days. For each species, the MES buffer from the plates was pooled (approximately 200 ml) and filtered through a Stericup® & Steritop®-HV 0.45 μm Durapore PVDF membrane Vacuum-Driven Filter Unit (MilliporeSigma, MA, U.S.A.) to capture cell-free filtrate. It was then concentrated using Amicon® Ultra-15 30K Centrifugal Filter Devices (MilliporeSigma, MA, U.S.A.). Proteins were quantified using a Pierce™ BCA Protein Assay Kit (Thermo Fisher Scientific, MA, U.S.A.) in conjunction with a BioMate™ 3S Spectrophotometer (Thermo Fisher Scientific, MA, U.S.A.).

For each species, 60 µg of proteins in 15 µl of the MES filtrate was diluted using NuPAGE™ LDS Sample Buffer (4×; Thermo Fisher Scientific, MA, U.S.A.), denatured at 95°C for 5 min, placed on ice for 3 min, and centrifuged at 15000×***g*** for 5 min. They were then separated on a NuPAGE 4–12% Bis-Tris Protein gel and stained with SimplyBlue™ SafeStain (Thermo Fisher Scientific, MA, U.S.A.). A total of 25 plugs (2 mm in diameter) of soluble proteins were excised from the vertical profile on the gel. Plugs were destained twice for 45 min each in 50% acetonitrile (ACN) containing ammonium bicarbonate (140 mM) and dehydrated in 100% ACN. Samples were rehydrated in trypsin digestion buffer containing ultrapure water, 10% ACN, 10 µg/ml of trypsin, and 40 mM of ammonium bicarbonate at room temperature (approximately 22°C) for digesting proteins to peptides. After 30 min, additional digestion buffer minus trypsin was added to completely submerge gel plugs. Digestion continued overnight at 37°C. Trypsin-digested peptides were extracted by the addition of 100 µl of water containing 0.1% trifluoroacetic acid (TFA), followed by two extractions using 100 µl of 50% ACN containing 0.1% TFA. Peptides were vacuum-dried in a DNA 110 SpeedVac (Thermo Fisher Scientific, MA, U.S.A.) and resuspended in 10 µl of 50% ACN containing 0.1% TFA.

### Mass spectrometry

Mass spectrometry (MS) analysis was performed on an Orbitrap Fusion Lumos (Thermo Fisher Scientific, San Jose, CA, U.S.A.) as previously described [40]. Positive ion static nanospray voltage was set to 2.4 kV and internal mass calibration was maintained with a user defined lock mass of a polydimethylcyclosiloxane ion at m/z 445.12003 [41]. MS survey scans were recorded in the Orbitrap at 120000 resolution over a mass range of 400–1600 m/z. The instrument was operated in top speed mode with a cycle time of 3 s. Monoisotopic peak selection for peptides was enabled. Dynamic exclusion was set to sample a peptide once only before placing its mass on an exclusion list for 20 s. Searchable peak lists were prepared using RawConverter [42] and saved as mascot generic format (mgf) files.

Proteins were identified using the Mascot search engine v. 2.6.0 (Matrix Science, London, U.K.). Predefined contaminant databases downloaded in Mascot Database Manager were used to identify prokaryotic and mammalian contaminants such as keratin, serum albumin, and excess trypsin (from peptide digestion). Reference protein datasets of *Che* strain CBS 138102 [43] and *Cps* strain CBS 139707 [44] were derived from their genome sequences [45,46] using CodingQuarray v. 2.0 [47]. Biological functions of proteins were preliminary annotated using Blast2GO v. 5.1 [48]. In order to match acquired spectra and identify pathogen proteins during the MS analysis, protein sequences in the datasets were concatenated using the following parameters: monoisotopic mass, parent ion tolerance of 5 ppm, fragment ion tolerance of 0.6 Da, ^13^C isotopes set to 2, peptide charge states of 1+, 2+, and 3+, trypsin as digesting enzyme with one missed cleavage allowed, and variable modifications of oxidation of methionine, N-terminal pyroglutamic acid from glutamic acid or glutamine. Scaffold v. 4.8.1 (Proteome Software Inc., Portland, OR, U.S.A.) was used to validate peptide and protein identification. Protein identifications were accepted if they could be established at greater than 95% probability and contained at least two identified peptides. Protein probabilities were assigned by the ProteinProphet algorithm (false discovery rate; FDR) in Scaffold [49]. The FDR values were adjusted to 0.4% for Peptide and 0.5% for Protein.

Three pairs of biological samples of *Che* and *Cps* were prepared for the MS analysis. The most robust set (No. 3) was selected for further analyses. Protein identifications were accepted if they could be established at ≥95.0% probability and contained at least two identified peptides. Moreover, prokaryotic and mammalian contaminants were excluded. Sequences of validated proteins of the sample set No. 3 in Scaffold v. 4.8.1 were exported to a FASTA data file for the following analyses.

To aid the assignment of subcellular localization (SL), all sequences were mapped to OrthoMCL clusters using the pre-configured workflow [50] at the EuPathDB Galaxy server [51]. One hundred and eighty-four proteins mapped to *NO_GROUP* or *unknown* by OrthoMCL were manually assigned to *NO_GROUP01–30* or *unknown01–63* clusters, respectively, by sequence similarity (Supplementary Table S1). Thereafter, all proteins were sorted by cluster. One sequence of a representative protein within each cluster was used to run BLASTP suite against the sequence database at NCBI [52] to confirm its Blast2GO annotation.

### Prediction of secreted proteins

**Figure 1:**
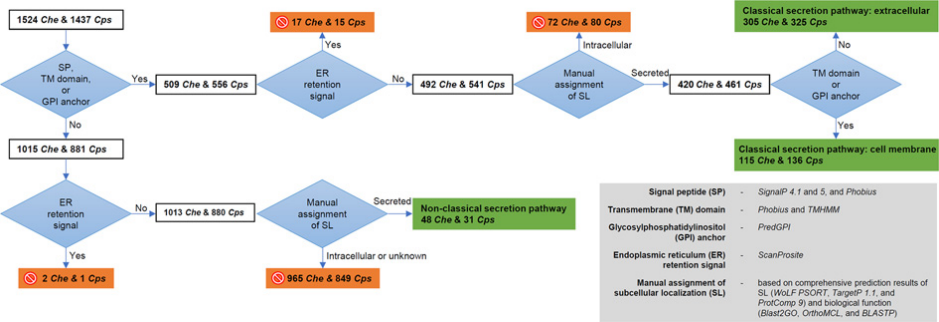
A pipeline based on manually validated prediction results of computational tools for identifying extracellular proteins in classical and non-classical secretion pathways and cell membrane proteins produced *in vitro* by *Che* and *Cps* These proteins are highlighted by a green background. Intracellular proteins that were eliminated from subsequent analyses are marked by a circle-backslash symbol on an orange background.

A modified pipeline [27] was used to identify secreted proteins of *Che* and *Cps* in classical and non-classical pathways (Figure 1). The presence of SP at the N-terminus was determined using SignalP 4.0 [53] and 5.0 [54] and Phobius 1.01 [55,56]. The number of transmembrane (TM) domains of each protein was predicted using Phobius [55,56] and TMHMM v. 2.0 [57]. The presence of a glycosylphosphatidylinositol (GPI) anchor was predicted using PredGPI [58]. The presence of an ER retention signal was predicted using ScanProsite [59]. Predictions from these tools were pooled together to give a list of SP-, TM-, and/or GPI domain-containing, ER retention signal-free proteins (Figure 1). SL of these proteins was predicted using the combined outputs of three SL prediction tools including WoLF PSORT [60], TargetP 1.1 [61], and ProtComp 9 (www.softberry.com/berry.phtml?topic=protcompan&group=programs&subgroup=proloc). Proteins that were assigned as ‘secreted’ by all three SL prediction tools were retained for further analyses. For proteins with contradictory SL predictions by the three tools, their localizations were manually assigned based on their biological functional annotations obtained from Blast2GO, OrthoMCL, and BLASTP (Supplementary Table S1). Proteins determined as being secreted in the classical pathway were then categorized into two groups, ‘cell membrane’ or ‘extracellular,’ correlating to the presence or absence of the TM domain and GPI anchor, respectively (Figure 1). The remaining proteins were assigned as ‘intracellular’ (Figure 1).

Among proteins without SP, TM domain, GPI anchor, or ER retention signals, their localizations were manually assigned based on their biological functional annotations of Blast2GO, OrthoMCL, and BLASTP (Supplementary Table S1). If their orthologs were found as being secreted by other fungi, they were assigned as extracellular proteins in the non-classical pathway (Figure 1). The others were assigned as ‘intracellular’ or ‘unknown’ (Figure 1).

### Functional analysis of secreted proteins

Three sets of protein sequences were taken forward for this functional analysis, including extracellular proteins in the classical secretion pathway, extracellular proteins in the non-classical secretion pathway, and cell membrane proteins. They were analyzed using the built-in functional analysis workflow in OmicsBox v. 1.1.164 (www.biobam.com/omicsbox). Briefly, sequences in each set were aligned to sequences in the NCBI Blast service (QBlast), mapped and annotated per their Gene Ontology (GO) term. They were also annotated using the EMBL-EMI InterPro web-service. The annotation results done by QBlast and InterPro were then merged in OmicsBox. Charts and statistics including GO counts were exported from OmicsBox.

### Prediction of putative effectors

Extracellular and cell membrane proteins of *Che* and *Cps* in the classical and non-classical secretion pathways were analyzed using EffectorP v. 1.0 [62] and 2.0 [63]. Proteins predicted as effectors of high confidence (Probability > 0.5) by both versions of the program were called as putative effector candidates in this study, for the purpose of minimizing the false positive likelihood [63]. Localization of putative effectors to the plant apoplast was predicted using ApoplastP [64].

## Results

### Protein sequences obtained from MS

The LC-MS/MS analysis resulted in a total of 7815 unique peptides including 3947 (51%) that were present in both *Che* and *Cps*, and 1724 (22%) and 2144 (27%) only present in *Che* or *Cps*, respectively. These peptides represented 2289 *Che* and 2055 *Cps* protein sequences, respectively. After excluding sequences of the proteins with a probability of <95.0%, represented by a singular peptide, and/or identified as contaminants, 1524 *Che* and 1437 *Cps* sequences (Supplementary Table S1) were retained for the further analyses.

### Extracellular and cell membrane proteins in the classical pathway

The presence of SP, TM domain, or GPI anchor motifs was determined for the 2961 protein sequences of *Che* and *Cps* (Figure 1). SignalP 4.1 found SP in 399 *Che* and 427 *Cps* sequences (Supplementary Table S1). SignalP 5 found SP in 401 *Che* and 432 *Cps* sequences (Supplementary Table S1). Phobius found SP in 455 *Che* and 480 *Cps* and at least one TM domain in 92 *Che* and 116 *Cps* sequences (Supplementary Table S1). TMHMM found TM domain(s) in 75 *Che* and 95 *Cps* sequences (Supplementary Table S1). PredGPI found GPI anchor motifs in 57 *Che* and 72 *Cps* sequences with ≥0.99 probability (Supplementary Table S1). Combining the above prediction results, 509 *Che* and 556 *Cps* SP-, TM domain-, and/or GPI anchor-containing sequences were retained to determine the presence of an ER retention signal (Figure 1). ScanProsite found the ER retention signal in 17 *Che* and 15 *Cps* sequences (Supplementary Table S1), which were excluded. SL for the remaining 492 *Che* and 541 *Cps* sequences were assigned using the collective SL prediction results of WoLF PSORT, TargetP, and ProtComp, and manual curation based on the bio-functional annotations of Blast2GO, OrthoMCL, and BLASTP. A total of 152 sequences (72 *Che* and 80 *Cps*) were unanimously predicted as intracellular by the three SL prediction tools or had orthologs that were verified as intracellular (Figure 1). The remaining 420 *Che* and 461 *Cps* sequences were determined as proteins belonging to the classical secretion pathway (Figure 1).

Depending on the presence of the TM domain(s) and/or GPI anchor, the 881 protein sequences in the classical secretion pathway were divided into two categories, namely extracellular proteins and cell membrane proteins. A total of 305 *Che* and 325 *Cps* sequences without TM domain(s) or GPI anchor were extracellular proteins (secretome), while the remaining 115 *Che* and 136 *Cps* sequences containing membrane-bound motifs were categorized as cell membrane proteins (Figure 1).

**Figure 2:**
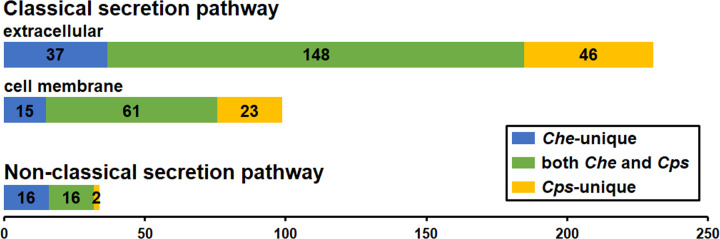
Orthologous cluster counts of *Che* and *Cps* in classical and non-classical secretion pathways

**Figure 3:**
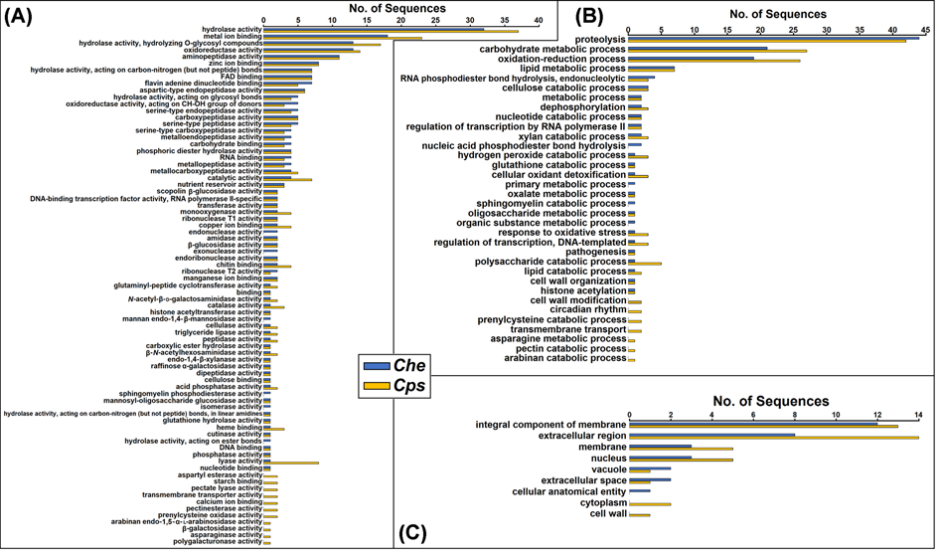
Direct GO counts in the MF (**A**), BP (**B**), and CC (**C**) categories among 306 *Che* and 326 *Cps* sequences of predicted extracellular proteins in the classical secretion pathway

The 630 extracellular proteins within the *Che* and *Cps* secretomes belonged to a total of 231 OrthoMCL clusters. The majority of the clusters (64%) were found in both species, while 16 and 20% were unique to *Che* and *Cps*, respectively (Figure 2). These proteins had predictions for diverse bio-functional roles in the three major GO categories, namely Molecular Function (MF), Biological Process (BP), and Cellular Component (CC). Within the MF category, 238 *Che* and 278 *Cps* protein GO terms were predicted to engage in approximately 70 distinct enzymatic activities such as hydrolysis, metal ion and nucleotide binding, and peptidase activities (Figure 3A). Proteins with GO terms predicting endonuclease activity, exonuclease activity, mannan endo-1,4-β-mannosidase activity, isomerase activity, and hydrolysis on ester bonds, were present only in *Che*, while those predicted to be involved with aspartyl esterase activity, starch binding, pectate lyase activity, transmembrane transporter activity, calcium ion binding, pectinesterase activity, prenylcysteine oxidase activity, arabinan endo-1,5-α-L-arabinosidase activity, β-galactosidase activity, asparaginase activity, and polygalacturonase activity occurred only in *Cps* (Figure 3A). Within the BP category, proteins in 125 *Che* and 156 *Cps* GO terms were predicted to engage in at least 30 biofunctions; the most common included proteolysis, metabolic processes, and oxidation-reduction processes (Figure 3B). Proteins unique to the *Che* dataset were predicted to conduct nucleic acid phosphodiester bond hydrolysis, primary metabolic processes, sphingomyelin catabolism, and organic substance metabolism. In contrast, proteins unique to the *Cps* dataset were predicted to be involved in seven processes, namely cell wall modification, circadian rhythm, prenylcysteine catabolism, transmembrane transport, asparagine metabolism, and pectin and arabinan catabolism (Figure 3B). Among 31 *Che* and 42 *Cps* GO terms within the CC category, 10 *Che* and 16 *Cps* were predicted to localize in the extracellular region, whereas 12 *Che* and 13 *Cps* were annotated as integral components of the membrane using OmicsBox (Figure 3C), despite the absence of membrane-bound peptides determined by other prediction tools.

**Figure 4:**
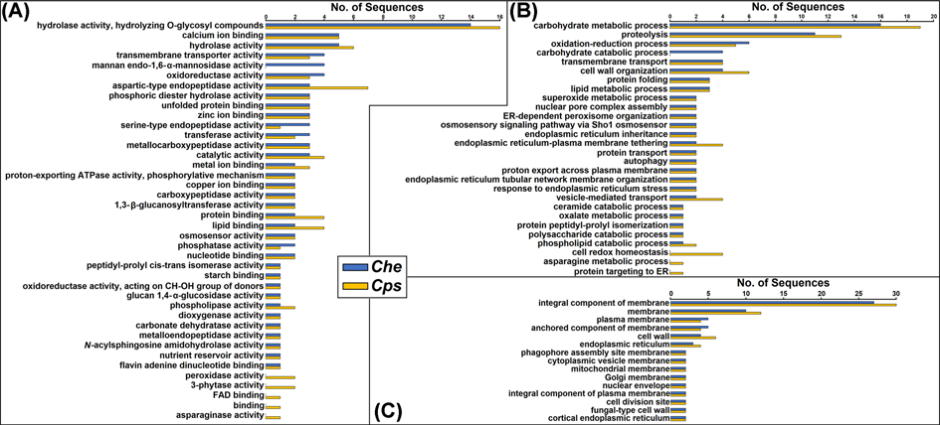
Direct GO counts in the MF (**A**), BP (**B**), and CC (**C**) categories among 115 *Che* and 136 *Cps* sequences of predicted cell membrane proteins

The 251 cell membrane proteins belonged to a total of 99 OrthoMCL clusters. Sixty-one clusters were found in both species, while 15 and 23 were unique to *Che* and *Cps*, respectively (Figure 2). For MF, protein members of both species were involved in approximately 40 functions (Figure 4A). Four proteins unique to the *Che* dataset were associated with mannan endo-1,6-α-mannosidase activity (Figure 4A). Seven proteins including peroxidase, 3-phytase, asparaginase activities, and those with FAD-binding were unique to the *Cps* dataset (Figure 4A). For BP, proteins of both species were predicted to engage in more than 25 processes of diverse metabolic pathways (Figure 4B). Proteins unique to the *Che* dataset were predicted to act in carbohydrate catabolic processes, while *Cps* proteins were predicted to act in cell redox homeostasis and asparagine metabolic processes (Figure 4B). For CC, most proteins were predicted as membrane components, while some were also part of the endomembrane system, such as nuclear envelope, ER, and vesicles (Figure 4C).

### Extracellular proteomes in the non-classical pathway

Excluding sequences containing the ER retention signal, SL of the remaining 1013 *Che* and 880 *Cps* proteins were assigned using the SL predictions of WoLF PSORT, TargetP, and ProtComp, and manual curation based on bio-functional annotations performed with Blast2GO, OrthoMCL, and BLASTP. A total of 48 *Che* and 31 *Cps* proteins were unanimously predicted by the three SL prediction tools or had orthologs that were verified as extracellular proteins. These 79 proteins found in the non-classical secretion pathway belonged to 34 OrthoMCL clusters. Sixteen clusters were present in both species, while 16 and two were unique to the *Che* and *Cps* datasets, respectively (Figure 2).

**Figure 5:**
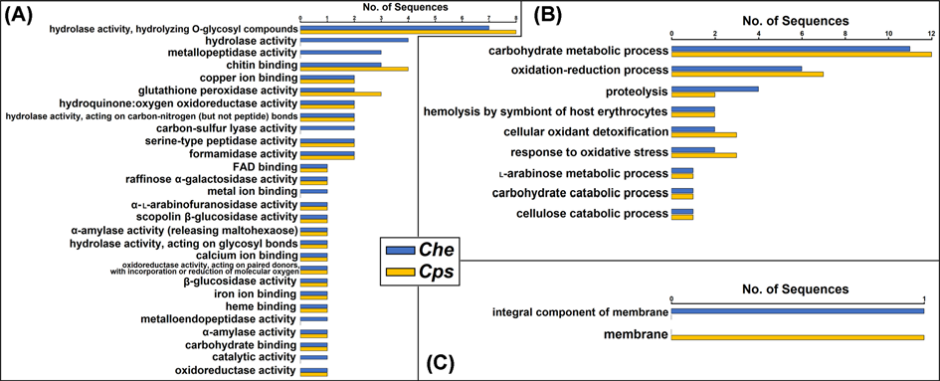
Direct GO counts in the MF (**A**), BP (**B**), and CC (**C**) categories among 49 *Che* and 31 *Cps* sequences of predicted extracellular proteins in the non-classical secretion pathway

For MF, extracellular proteins of both species in the non-classical secretion pathway were predicted to play roles in hydrolysis, peptization, and compound binding, while only proteins belonging to the *Che* dataset were predicted to have specific functions such as metallopeptidase and carbon-sulfur lyase activities (Figure 5A). For BP, these proteins were predicted to have a role in metabolic processes, with the top three carbohydrate and oxidation-reduction processes and proteolysis (Figure 5B). For CC, one protein of each species was predicted as a membrane component by OmicsBox (Figure 5C). However, no membrane-bound motifs were found in these two protein sequences using other prediction tools.

### Putative effectors of *Che* and *Cps*

Putative effectors were predicted among the 630 extracellular proteins and 251 cell membrane proteins in the classical secretion pathway, as well as the 79 extracellular proteins in the non-classical pathway. For extracellular proteins in the classical pathway, 52 *Che* and 48 *Cps* proteins were predicted as putative effectors (Supplementary Table S1). For cell membrane proteins, three *Che* and five *Cps* proteins were predicted as putative effectors (Supplementary Table S1). For extracellular proteins in the non-classical pathway, nine *Che* and seven *Cps* proteins were predicted as putative effectors (Supplementary Table S1). These 124 proteins belonged to 52 OrthoMCL clusters, including 15 *Che*- and 11 *Cps*-unique clusters, and 24 that were present in both species (Table 2).

**Table 2:** List of putative effectors identified from extracellular and cell membrane proteins of *Che* and *Cps*

Species	Apoplastic location	OrthoMCL cluster^1^	Sequence(s)	Localization	Biological function	GO name^2^
*Che*	Apoplastic	OG5_163464	CUFF.12275.1.5	Extracellular, non-classical	Glutathione-dependent formaldehyde-activating enzyme	F: carbon-sulfur lyase activity
			TCONS_00007935.1169			
		OG5_188927	CH.00496	Extracellular	Hypothetical protein DL765_009141	P: proteolysis; F: metallopeptidase activity
		**unknown004**	CH.01838	Extracellular	Secreted protein	n.a.
		**unknown005**	CH.03559	Extracellular	Hypothetical protein AK830_g2578	n.a.
			CPS.08498			
		**unknown015**	CH.08044	Extracellular	Hypothetical protein Micbo1qcDRAFT_206154	n.a.
		**unknown016**	CH.08408	Extracellular	Hypothetical protein TRIVIDRAFT_131328, partial	n.a.
			CPS.07529			
		**unknown030**	CUFF.11335.1.0	Extracellular	Major allergen alt	n.a.
		**unknown033**	CUFF.147.1.8	Extracellular	Small secreted cysteine-rich protein (SSCRP)	n.a.
	Non-apoplastic	OG5_127576	CUFF.6398.1.0	Extracellular	Related to plant PR-1 class of pathogen related proteins	n.a.
		OG5_141094	TCONS_00003137.420	Cell membrane	γ interferon inducible lysosomal thiol reductase	C: integral component of membrane
		**OG5_159533**	CUFF.189.1.2	Extracellular, non-classical	Hypothetical protein AK830_g9984	n.a.
		**unknown006**	CH.03657	Extracellular	—NA—	n.a.
		**unknown007**	CH.03681	Extracellular	Hypothetical protein CC84DRAFT_1263931	n.a.
		**unknown018**	CH.09694	Extracellular	—NA—	n.a.
		**unknown019**	CPS.00858	Extracellular	—NA—	n.a.
*Che*; *Cps*	Apoplastic	NO_GROUP012	CPS.02420	Extracellular	Putative cutinase 1	F: carboxylic ester hydrolase activity
		NO_GROUP019	CUFF.7628.1.1	Extracellular	Chorismate mutase, type II	C: membrane; C: integral component of membrane; P: chorismate metabolic process
			TCONS_00009802.394			
		**NO_GROUP021**	CUFF.218.1.0	Cell membrane	Hypothetical protein AK830_g2507	n.a.
			TCONS_00003574.146			
		OG5_149850	CUFF.30.1.0	Extracellular	Cutinase; carbohydrate esterase family 5 protein	C: extracellular region; P: pathogenesis; F: cutinase activity
			TCONS_00005083.204			
		OG5_149851	CUFF.1699.1.1	Extracellular	Guanyl-specific ribonuclease F1	F: RNA binding; F: endoribonuclease activity; F: ribonuclease T1 activity; P: RNA phosphodiester bond hydrolysis, endonucleolytic
			TCONS_00005542.18			
		OG5_152723	CUFF.12308.1.2	Extracellular	Eliciting plant response-like protein; protein SnodProt1	n.a.
			CUFF.8119.1.1			
			CUFF.9063.1.6			
			TCONS_00000004.4			
			TCONS_00006271.15			
			TCONS_00006369.287			
		OG5_155754	CH.07302	Extracellular	Cell wall protein PhiA; hypothetical protein CEP54_009425	n.a.
			CUFF.6792.1.0			
			TCONS_00005539.0			
			TCONS_00009210.105			
			TCONS_00014055.66			
			TCONS_00014195.42			
		OG5_159298	CH.02115	Extracellular	gdsl-like lipase acylhydrolase	P: metabolic process; F: hydrolase activity, acting on glycosyl bonds
		**OG5_173175**	CUFF.2807.1.0	Extracellular	Predicted protein	n.a.
			TCONS_00001797.825			
		**OG5_188749**	CH.09023	Extracellular	UPF0311 protein; hypothetical protein Micbo1qcDRAFT_106863, partial	n.a.
			CPS.05761			
		**OG5_203284**	CH.06526	Extracellular	Hypothetical protein NECHADRAFT_122703; CDV36_000878	n.a.
			CH.09635			
			CPS.00358			
		unknown014	CH.07830	Extracellular	Heat-labile enterotoxin IIA, A chain	C: extracellular space; P: pathogenesis; F: toxin activity
			CPS.07297			
		**unknown036**	CUFF.5085.1.1	Extracellular	Hypothetical protein AK830_g8252	n.a.
			TCONS_00004137.536			
	Non-apoplastic	**NO_GROUP015**	CUFF.10007.1.0	Extracellular	Hypothetical protein FIE12Z_11595	n.a.
			TCONS_00012764.424			
		OG5_126711	CUFF.12042.1.4	Extracellular, non-classical	Glutathione peroxidase	F: glutathione peroxidase activity; P: response to oxidative stress; P: oxidation-reduction process; P: cellular oxidant detoxification
			TCONS_00003797.179			
			CPS.01909			
		OG5_127142	CUFF.2297.1.9	Extracellular, non-classical	Cytochrome b5; putative progesterone binding protein	n.a.
			TCONS_00008509.1189			
		OG5_127452	CUFF.8752.1.7	Cell membrane	Peptidyl-prolyl *cis-trans* isomerase b	P: protein peptidyl-prolyl isomerization; F: peptidyl-prolyl cis-trans isomerase activity
		OG5_129624	TCONS_00011054.33	Extracellular	Dienelactone hydrolase	F: hydrolase activity
		OG5_159304	CH.00799	Extracellular, non-classical	Aegerolysin aa-Pri1	P: hemolysis by symbiont of host erythrocytes
			CPS.02349			
		**OG5_211107**	CUFF.6707.1.1	Extracellular	Hypothetical protein AK830_g2470	n.a.
			TCONS_00012290.703			
		**OG5_233008**	CH.08973	Extracellular	Hypothetical protein UCREL1_198	n.a.
			CPS.05928			
		unknown012	CH.05735	Extracellular	Carbohydrate-binding module family 13 protein	F: amidase activity; F: *N*-acetylmuramoyl-_L_-alanine amidase activity; P: peptidoglycan catabolic process; F: carbohydrate binding
			CPS.02503			
		**unknown028**	CPS.08330	Extracellular	Hypothetical protein BN4615_P9102	n.a.
			CUFF.2832.1.2			
		**unknown044**	CUFF.9764.1.3	Extracellular	Hypothetical protein CGGC5_9092	n.a.
			TCONS_00009751.285			
*Cps*	Apoplastic	OG5_126613	CUFF.8366.1.3	Cell membrane	Thioredoxin-like protein (probable thioredoxin)	P: cell redox homeostasis
			TCONS_00008072.249			
		OG5_169371	CH.01131	Extracellular	Filamentous hemagglutinin/adhesin	n.a.
			CPS.02832			
		**unknown047**	CPS.03762	Extracellular	Hypothetical protein Micbo1qcDRAFT_206154	n.a.
		**unknown050**	PGN.14881	Extracellular	Hypothetical protein AK830_g8252	n.a.
		**unknown061**	TCONS_00009299.315	Extracellular	—NA—	n.a.
	Non-apoplastic	OG5_149749	CUFF.5974.1.0	Extracellular	Related to serine proteinase inhibitor IA-2	n.a.
			TCONS_00002105.598			
		OG5_168898	CH.04048	Extracellular	Putative pectate lyase F	C: extracellular region; F: pectate lyase activity
			CPS.06721			
		**OG5_233276**	CH.08975	Extracellular	Hypothetical protein AK830_g4145	n.a.
			CPS.01629			
		**unknown046**	CPS.03447	Extracellular	—NA—	n.a.
		**unknown048**	CPS.04723	Extracellular	—NA—	n.a.
		**unknown058**	TCONS_00001477.21	Extracellular	Hypothetical protein VFPFJ_02932	n.a.

1 Names of clusters containing putative effectors without clear biological functions or GO names are in bold.

2 n.a., not available.

With regard to their biological functions, approximately 46% of the putative effectors had undetermined functions or GO names using OmicsBox. The others had diverse functions (Table 2) such as hydrolysis (OrthoMCL clusters: OG5_163464, NO_GROUP012, OG5_159298, OG5_129624), pathogenesis (OG5_149850, OG5_152723, OG5_155754, unknown014, OG5_168898), proteolysis and peptization (OG5_188927), cutinase activity (NO_GROUP012, OG5_149850), and response to oxidative stress (OG5_126711).

## Discussion

Understanding the biology and pathogenesis of the boxwood blight pathogens is fundamental to the protection of horticultural and landscape industries, established gardens, and natural ecosystems from the devastation caused by this disease. Boxwood is a plant of economic and cultural importance. With an estimated value of $170 million in U.S.A. alone [1,2], boxwood production is a critical asset to the ornamental plant nursery and landscape industries. Boxwood is also the backbone landscape plant of countless heritage sites worldwide, including Mount Vernon, Colonial Williamsburg, and Monticello in U.S.A. In the present study, a comparative proteomic analysis was carried out to enhance our knowledge on the extracellular proteins, including pathogenesis-related effectors produced by *Che* and *Cps*. The results of the present study have several implications.

Extracellular proteins produced by both boxwood blight pathogens are involved in a diversity of molecular functions and processes that may be critical to pathogen growth and infection. Comparing *Che* and *Cps*, similar GO counts were observed in the MF and BP categories of the extracellular proteomes (Figures 3 and 5). This suggests that these two closely related species generally produced similar proteins and have comparable metabolic processes under the *in-vitro* condition in the present study. Extracellular proteins of *Che* and *Cps* identified in the present study were predicted to be involved in a variety of hydrolase activities, such as acting on glycosyl compounds, carbon-nitrogen bonds, and peptide bonds (Figures 3 and 5). They were also active in proteolysis, carbohydrate metabolism, and oxidation-reduction processes (Figures 3 and 5). Although proteins that play roles in MF and BP may not directly be involved in pathogenesis, they are expected to function in ecological adaptation, response to environmental stresses, and cell metabolism and growth, which may be prerequisites for pathogenesis. For example, 958 expanded gene families were found in another member of the genus *Calonectria*, *C. pseudoreteaudii* based on *in-silico* genomic analysis [35]. The expansion of these genes indicated active host adaptive processes and backbone enzyme production in *C. pseudoreteaudii* [35]. Whether the boxwood blight pathogens have also expanded these gene families and related proteins warrant a comparative study, including other species in the genus *Calonectria* and the Nectriaceae family, such as *Pseudonectria foliicola*, the causal agent of Volutella blight of boxwood.

Cell membrane proteins of *Che* and *Cps* are not simply an integral component of the cell structure, but also play vital molecular and biological roles. The majority of 115 *Che* and 136 *Cps* cell membrane proteins predicted using the bioinformatics workflow (Figure 1) were also annotated as components of the plasma membrane or cell wall using OmicsBox (Figure 4C). Approximately eight protein members of each species were associated with the production of cytoplasmic vesicles and that of mitochondrial, Golgi, and nuclear membranes as annotated by OmicsBox (Figure 4C). These annotations appeared to be contradictory to the prediction results using the workflow (Figure 1), although these proteins could localize to the plasma membrane by moving through the intracellular membranes. The exact SL of these proteins warrants further validation. This can be achieved using fluorescent-tagged proteins followed by histological examination in a homologous or heterologous system. In addition to being important structural components, cell membrane proteins of the boxwood blight pathogens may also have vital roles in cell signaling, regulation, and metabolism. Cell membrane proteins act as enzymes carrying out a variety of hydrolase, oxidoreductase, transferase activities, as well as transmembrane transport (Figure 4). Although the exact significance of their roles is unclear, they could likely involve the degradation of plant cells and the export of other pathogenesis-related proteins. Due to their consistent presence in the cell membrane and critical role in fungal survival and pathogenesis, cell membrane proteins of fungal pathogens are targets often used for developing antibodies and immune-therapies for human diseases [27,65]. For the boxwood blight pathogens, antibody-based diagnostic assays targeting proteins identified in this study are currently under development. Specifically, one high-abundance protein unique to *Che* and *Cps* was identified among those in the classical secretion pathway and has been targeted for further study. Antibodies have been raised against the protein and found to react with proteins extracted from lesions on *Buxus* leaves infected with *Che* and *Cps* (D.G. Luster, personal communication).

More than 120 putative effectors of *Che* and *Cps* were predicted in the present study, while their exact roles in the pathogenesis of boxwood blight warrant further analyses. Many of the putative effectors of *Che* and *Cps* had predicted functional similarity to effectors documented in other plant pathosystems. Among the putative effectors present in both species, the two largest OrthoMCL clusters are OG5_152723 and OG5_155754. Each of these two clusters contains six apoplastic putative effectors (Table 2). The OG5_152723 cluster contains SnodProt1 and its homologs known as pathogenesis response elicitors and virulence-related effectors previously found in many important plant pathogens, such as the glume blotch fungus *Phaeosphaeria nodorum* [66], the blast fungus *Magnaporthe grisea* [67], the Verticillium wilt fungus *Verticillium dahliae* [68], and the Fusarium head blight fungus *Fusarium graminearum* [69,70]. Cluster OG5_155754 contains cell wall protein PhiA and its homologs (Table 2), which are known for their critical roles in conidia formation and stress response of *Aspergillus* species [71,72] and *Fusarium oxysporum* [73]. Two clusters, OG5_149850 and NO_GROUP012 include cutinase enzymes that can facilitate pathogen ingress and infection by hydrolyzing plant cutin [74,75]. In addition, homologs of chorismate mutase [76,77] represented by cluster NO_GROUP019, guanyl-specific ribonuclease by OG5_149851 [78], gdsl-like lipase acylhydrolase [62] by OG5_159298, and dienelactone hydrolase [79] by OG5_129624 were reported as effectors of other plant pathogens, while those of heat-labile enterotoxin IIA represented by cluster unknown014 were produced by human-pathogenic *Escherichia coli* [80]. These homologs of previously reported effectors may play major roles in the boxwood blight disease and warrant functional validation. Additionally, more than half of the putative effectors had either undetermined functions or GO names as predicted by OmicsBox, or annotated functions not previously reported for any fungal effectors (Table 2). While it is unlikely that every predicted effector plays a role in infection, the findings here reveal the current gap in our knowledge on the pathogenesis of the fungi causing boxwood blight and provides a repository of potentially novel effectors to be analyzed in future studies. The functions of individual predicted effectors can be experimentally validated upon the establishment of a transformation system for *Che* and *Cps* or using a heterologous system to characterize protein function. Furthermore, putative effectors identified in this study may be useful for identifying resistance genes in resistant cultivars and nonhosts. For example, high-throughput screenings for resistance genes using putative effector candidates have been performed for other plant pathosystems, including late blight of potato caused by *Phytophthora infestans* [81,82] and downy mildew of lettuce caused by *Bremia lactucae* [83]. As no curative fungicide has been found and durable host resistance may be the only long-term solution for controlling boxwood blight, the identification of *Che* and *Cps* putative effectors in this study will potentially contribute to the screening for resistance genes and downstream breeding programs of resistant boxwood cultivars.

While 712 extracellular proteins and 251 cell membrane proteins were identified in the present study using *in-vitro* experimental materials, it is important to continue the discovery of *Che* and *Cps* proteins using other approaches. The experimental proteomes produced *in vitro* should be considered as only part of the entire extracellular proteomes of *Che* and *Cps*. Many other proteins could be secreted exclusively *in planta*. However, to take the *in-planta* approach, a reference protein dataset derived for a boxwood host genome is a prerequisite to match the acquired spectra and distinguish pathogen versus host proteins during the MS analysis. At present, no *Buxus* genomes are available to generate such a reference dataset. Additionally, different fungal structures, depending on their roles in the life cycles of the pathogens, may secrete distinct proteins to accomplish various biological functions. For *Che* and *Cps*, mycelia are responsible for vegetative growth and expansion, while microsclerotia [84] formed by specialized mycelia can survive under adverse environmental conditions for at least 5 years [85]. When environmental conditions are favorable, microsclerotia sporulate to form sporodochia, each containing masses of conidia, the asexual spores that initiate infection. In the present study, proteins were mainly produced by mycelia submerged in MES buffer. It will be interesting to compare the extracellular proteomes of the present study with those exclusively produced by conidia and microsclerotia, which potentially contain additional key proteins related to fungal pathogenicity and response to stresses, respectively, although separating and accumulating enough material from the individual microstructures may pose a particular challenge.

In the present study, we used a bioinformatics approach to predict extracellular proteins in experimental samples prepared *in vitro*. We note that previous studies applied an *in-silico* approach to predict fungal secretomes, based entirely on genome sequences [27,31,35]. Similar *in-silico* analytical predictions of *Cps* and *Che* secretomes based on their genome sequences are ongoing (J. Crouch, personal communication). It will be interesting to compare those with *in vitro*-produced extracellular proteins identified in the present study to better understand protein expression of the boxwood blight pathogens under different conditions.

## Supplementary Material

Supplementary Table S1

## Data Availability

All data are provided. Original genome sequence and protein datasets are cited and links are provided in the ‘References’. Analyzed data are provided in the supplementary table.

## Abbreviations

ACN: acetonitrile
BP: biological process
CC: cellular component
*Che*: *Calonectria henricotiae*
*Cps*: *Calonectria pseudonaviculata*
ER: endoplasmic reticulum
GO: gene ontology
GPI: glycosylphosphatidylinositol
GYET: glucose-yeast extract-tyrosine
LC-MS/MS: liquid chromatography-tandem mass spectrometry
MES: 2-(N-morpholino)ethanesulfonic acid
MF: molecular function
MS: mass spectrometry
SL: subcellular localization
SP: signal peptide
SSCP: small secreted cysteine-rich protein
TFA: trifluoroacetic acid
TM: transmembrane
